# Cuteness or Coolness—How Does Different Anthropomorphic Brand Image Accelerate Consumers’ Willingness to Buy Green Products?

**DOI:** 10.3389/fpsyg.2021.599385

**Published:** 2021-08-31

**Authors:** Yue Lu, Yunxiao Liu, Le Tao, Shenghong Ye

**Affiliations:** ^1^School of Business Administration, Guangdong University of Finance and Economics, Guangzhou, China; ^2^School of Management, Jinan University, Guangzhou, China

**Keywords:** green consumption, anthropomorphism, advertising appeal, brand trust, sustainability, purchase intention

## Abstract

Green consumption is an important component of environmental protection behavior. The behaviors of individual consumers are having unprecedented impacts on the sustainable development of a green society. Previous research has discussed how anthropomorphic beneficiaries of environmental behavior (e.g., nature/earth) impact green consumption behavior and compared the influence of anthropomorphic presence and absence on consumers. However, few have examined the impact of different types of anthropomorphic carriers with environmental benefits (e.g., green product/brand) on consumers. This research explores the matching effects on the willingness of consumers to buy green products between the anthropomorphic image of the brand (cute vs. cool) and advertising appeals (self-interest vs. altruism); in addition, the underlying mechanisms of matching effects are revealed. The results show that, under the self-interested advertising appeal, the cool anthropomorphic image can lead to higher purchase intention of green products due to the mediating role played by the brand capacity trust. However, when exposed to altruistic advertising appeal, the cute anthropomorphic image can enhance brand goodwill trust of consumers and make consumers more willing to buy green products. Finally, this paper discusses the contributions and limitations.

## Introduction

Environmental problems are related to the sustainable development of mankind. On the one hand, the human demand for environmental resources is increasing rapidly, and resources of the earth are overexploited; on the other hand, the environmental pollution caused by human activities is becoming more and more serious. In the relationship between humans and the environment, humans play an important role. Environmental problems are largely caused by human behavior (Dong et al., 2017), especially consumption behavior. If people convert traditional marketing behavior into green consumption, these problems will be alleviated. Although the relationship between marketing and green consumption has attracted the attention of people (Anderson and Cunningham, 1972; Kilbourne and Beckmann, 1998; Kotler, 2011), scholars of this field are still calling for studies on predictors of green consumption (Menon and Menon, 1997; Mick, 2006; White et al., 2019). By paying attention to the green consumption behavior of consumers in marketing practice, marketers can find new green consumption territory and finally expand the market for the long-term mutual benefit of enterprises and the ecological environment.

Marketing communication (green advertising, brand image, etc.) is an important means to promote the success of green product marketing. For example, many studies suggested that green product advertisements often focus on highlighting one of the dual attributes of green products (self-interest and altruism) to promote consumer purchase (Schuhwerk and Lefkoff-Hagius, 1995; Peloza et al., 2013; Kareklas et al., 2014; Yang et al., 2015). In addition to considering the attribute of green products, the brand attribute of green products cannot be ignored. Some studies show that brand-perceived value, brand trust, and brand awareness can all positively predict the purchase intention of consumers to green products (Rahbar and Abdul Wahid, 2011; Alshura and Zabadi, 2016; Ranjan and Kushwaha, 2017). Consumers often lack trust in products and advertisements that claim to be environmentally friendly, even for those consumers who have a high degree of environmental concern or master certain environmental knowledge. They will also doubt the environmental protection claims and motives of enterprises, which hinders them from further adopting green consumption behaviors (Tung et al., 2012; Nittala, 2014). Therefore, in the marketing of green products, it is particularly important to improve the trust of consumers in green products and brands through appropriate means of marketing communication. Some research show that the application of anthropomorphic images helps to enhance the trust of consumers in products and brands. Specifically, anthropomorphic product design and brand image can reduce the perceived risk of consumers (Jarvenpaa and Leidner, 1999; Kim and McGill, 2011); the lower the perceived risk, the higher the brand trust (Lau and Lee, 1999); thus, the inner conflict during purchase is reduced, and the purchase intention is strengthened (Hur et al., 2015).

**FIGURE 1:**
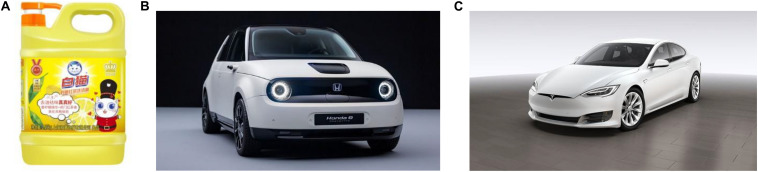
Environmental detergent with cute cat anthropomorphic image **(A)** and new energy environmental vehicles’ front-face modeling with cute anthropomorphic elements **(B)** and cool anthropomorphic elements respectively **(C)**.

As an effective communication element, anthropomorphism has become vitally important not only in the field of traditional marketing but also in the practice of environmental protection, including green consumption (Waytz et al., 2010). Previous studies have focused on the impact of environmental protection beneficiaries (nature and animals) as anthropomorphic objects. For example, various environmental activities often personify the earth as “Mother Earth.” At present, anthropomorphic elements have been used in the brand image and appearance design of some green products, for example, White Cat’s latest British black tea flavor environmental detergent uses the cute white cat anthropomorphic image (as shown in the lower right corner of Figure 1A), and some new energy environmental vehicles are designed with anthropomorphic elements in front-face modeling, such as a cute electric car of Honda (Figure 1B) and cool Model S series of Urban EV Tesla (Figure 1C).

It is worth noting that the anthropomorphic images of these green products use two popular subculture elements—cuteness and coolness. They can all serve as the direction for the personified image of the brand. Cute itself is a specific form of anthropomorphism (Epley et al., 2007; Chang et al., 2017), while cool can also be used as a personality trait to describe people, and existing studies have integrated it into brand personality (Warren et al., 2019), so it can also be used as a specific form of brand personification. More importantly, extant work often concentrates on the influence of anthropomorphism presence or absence on the attitude and behavior of consumers in the context of green consumption, but the research on whether different anthropomorphic image types can produce different effects is quite limited, so which element can achieve better marketing promotion effect in what situation is worth investigating.

Extending research show that the corresponding marketing communication effect playing by different anthropomorphic images is largely affected by context factors (Puzakova et al., 2013; Puzakova and Kwak, 2017; Reavey et al., 2018). As mentioned above, enterprises producing green products design a brand image through cuteness and coolness in ways that extend different types of a brand anthropomorphic image. Thus, whether these different anthropomorphic images of the brand will have different effects on green product marketing promotion (e.g., matching with different advertising appeal types) is to be discussed in this paper.

## Literature Review and Hypotheses Development

### Brand Anthropomorphic Image Types: Cute vs. Cool

Academically, the concept of cuteness was first proposed by Austrian zoologist Konrad Lorenz to generalize a series of visually perceived infant appearance or behavior characteristics (Lorenz, 1943), that is, “kindchenschema cuteness.” Because cuteness is often associated with those young children or things, thanks to this positive stereotype, the perception of cuteness can induce consumers to generate positive inferences such as innocence, kindness, and honesty (Keating et al., 2003). From the aspect of anthropomorphic marketing, elements of cuteness are considered to be a concrete way of anthropomorphic image design for brands or products (Epley et al., 2007; Chang et al., 2017). Based on consumer perception, cuteness is a positive perception that will make consumers feel compassion (Hellén and Sääksjärvi, 2013). Cuteness can be a unique marketing tool. That is to say, through product design, brand image, market positioning, and other ways, enterprises make consumers feel cute to their products or brands to initiate the positive evaluation of consumers of these products or brands, and thus promote the purchasing behavior of consumers (Desmet et al., 2001; Norman, 2004; Chitturi, 2009). For example, the study of Windhager et al. (2008) showed that, compared with the general automobile appearance features, cars with cute infant features would be perceived as friendlier and more easygoing, and the products would be evaluated correspondingly higher. Cheok (2010) also found that product image spokesmen with some infant characteristics can often lead consumers to have a higher evaluation of the product.

Furthermore, one common aspect of the research on cuteness is that people decide to engage in prosocial behavior, for instance, consumers are willing to help others or participate in donations and waste recycling (Bellfield et al., 2011; Miesler et al., 2011; Sherman and Haidt, 2011; Chang, 2012; Wang and Anirban, 2015; Wang et al., 2017). A cuteness schema can enhance the altruistic tendency of people and even make people start thinking about the interest of enterprises on account of the empathic tendency stimulated by cuteness (Sherman et al., 2009; Miesler et al., 2011). Similarly, research demonstrates that the response of people to cuteness is a kind of moral emotion because the sociality induced by cuteness can expand the moral circle of people and urge people to give more moral care (Sherman and Haidt, 2011). The willingness of consumers to buy green products is also affected by the diverse brand image. Pieces of research have classified brand anthropomorphic images, such as servant and partner (Aggarwal and McGill, 2012; Kim and Kramer, 2015; Alvarez and Fournier, 2016), competence and warmth based on the stereotype model (Aaker et al., 2010). The cute image may also evoke a perception of warmth, so there is a link between the cute and the warm stereotype (Sprengelmeyer et al., 2009). However, it is worth noting that the consumption behavior of green products is different from the general consumption behavior, which has a certain degree of moral and prosocial attributes (Griskevicius et al., 2010; White and Simpson, 2013). Although both cute and ordinary warm images (which do not give consumers a high perception of cuteness) can give consumers a warm perception, the difference is that consumers may not be able to generate such strong moral emotions for warm anthropomorphic images. Therefore, this study will mainly investigate the role of the cute brand anthropomorphic image in the purchase of green products, instead of the warm brand anthropomorphic image.

Likewise, coolness is also an increasingly common subcultural phenomenon (Gerber and Geiman, 2012). In the pursuit of personalized consumption of today, cool products and brands are always sought after by consumers, especially for young consumers who want to look cool (Nancarrow et al., 2002; Belk et al., 2010; Warren et al., 2018). More and more enterprises are trying to make their products, services, and brands cool to make consumers have perceived coolness. Specifically, enterprises establish cool brand images, employ cool brand spokesmen, design cool advertisements, and integrate cool elements into products or services (Rahman, 2013; Sundar et al., 2014). From the perspective of personality trait research, coolness is defined as a complex of personality traits that contain one or more personality traits (Belk, 2006; Dar-Nimrod et al., 2012). Studies have shown that coolness is positively correlated with traits such as Big Five, self-orientation, and emotional stability (Dar-Nimrod et al., 2018). As a style of personality characteristics, coolness can also be used for brand personification and shaping the brand personality of coolness (Warren et al., 2019). From the perspective of consumer perception, coolness is a kind of emotion or perception with both hedonism and functionality (Runyan Rodney et al., 2013). Coolness can be a sort of positive evaluation of products or services that include judgments of consumers on uniqueness, attractiveness, subculture, and other aspects (Sundar et al., 2014). A series of studies have shown that making consumers feel cool can significantly affect the psychology and behavior of consumers. According to the research results of Im et al. (2015), cool perception can enhance the hedonistic value of new products and then positively influence consumer attitude. Warren et al. (2018) found that cool brand spokesmen can enhance the favorable degree of the brand to consumers. In addition, the brand can enhance attitude of consumers toward the brand by displaying its cool personality, and thus increase the purchase intention of consumers to the products of the brand (Warren et al., 2019).

More importantly, existing studies have shown that initiating cool perception of consumers also increases the likelihood of taking prosocial behaviors (Nancarrow et al., 2002; Bird and Tapp, 2008; AS Mohiuddin et al., 2016). While coolness has features such as rebellion and self-centeredness that appear to contradict prosocial behavior, studies have found that prosocial behavior and coolness may be perceived as signals of maturity and social justice by consumers (Nancarrow et al., 2002; Bird and Tapp, 2008). Meanwhile, some dimensions of coolness are positively related to prosocial values (Dar-Nimrod et al., 2018). Similarly, consumers are more willing to take positive word-of-mouth communication behavior in virtue of the initiation of cool conception (Moldovan et al., 2011).

To sum up, both cuteness and coolness could make consumers have positive perceptions and be used as the anthropomorphic image development direction. According to the above studies, cuteness and coolness are still a pair of concepts that can be distinguished and relative. In particular, cuteness tends to evoke impressions of innocence, naivety, kindness. and warmth of consumers (Keating et al., 2003), while coolness often implies a high degree of autonomy, maturity, deviation from conventional norms, and innovation (Dar-Nimrod et al., 2018). The relationship between cuteness and coolness is similar to the warmth-competence dimension of the stereotype content model. Cuteness and coolness also promote prosocial behavior in certain contexts, so they can be used in green consumption studies. For other anthropomorphic categories (e.g., companion vs. servant and warmth vs. competence), there is little research to suggest that they can also play a positive role in promoting prosocial behavior, except for warm anthropomorphic images, which show that anthropomorphic characteristics of money can enhance warmth perception and promote charitable giving (Zhou et al., 2019). Moreover, dropping contextual factors to compare the effects of different anthropomorphic types on consumer cognition and decision-making would be difficult (Williams et al., 2015; Han et al., 2019; Ketron and Naletelich, 2019; Zhu et al., 2019). This paper aims to compare the roles of the cute brand anthropomorphic image and the cool brand anthropomorphic image in improving purchase intention under the green consumption context based on the characteristics of cuteness and coolness.

### The Advertising Appeal Type: Self-Interested vs. Altruistic

Advertising appeal is the content that should be emphasized in advertising (Holbrook and Batra, 1987). In commodity advertising, it refers to a variety of creative ways to capture the attention of the advertising audience, then transmitting the beneficial attributes of a product or service to stimulate their potential demand for this product or service and affect their attitude toward products or services and promote the occurrence of corresponding buying behavior ultimately (Schuhwerk and Lefkoff-Hagius, 1995). Appropriate green product advertising appeals can positively affect the attitudes of consumers toward green products and enhance the willingness of consumers to buy. The commonly used appeals in green product advertising include abstract appeal and concrete appeal, rational appeal and perceptual appeal, self-interest appeal and altruistic appeal, guilt appeal, and the gain-and-loss messages framework. To better explore the matching effect of green product advertising appeals and brand anthropomorphic image type on improving purchase intention, this research adopts altruistic and self-interest green product advertising appeal classification as well because green products themselves have altruistic and self-interested attributes (Mazar and Zhong, 2010).

In the field of green consumption, considerable studies are based on the assumption that consumers believe that altruism and self-interest cannot coexist and customarily value utility attributes (whether the efficacy is powerful or not) rather than ethics (whether it is beneficial for environmental protection). But as a matter of fact, the finite moral hypothesis and the finite self-interest hypothesis, which take into account the dual properties in the framework of green consumption, are closer to reality (White et al., 2019). The finite moral hypothesis and the finite self-interest hypothesis hold that individuals will consider both personal interests and social welfare in their behavioral decisions (Schlaile et al., 2018; White et al., 2019). However, consumers will focus primarily on either personal or social interests, which will be guided and influenced by the advertising appeal. Altruistic advertising appeal that is beneficial to the environment highlights the ethical attributes of green products and pays attention to the interests of the whole ecological environment and society to increase the prosocial tendency of consumers to a certain extent. Self-interested advertising appeal that is beneficial to individuals invariably highlights the utility attributes of green products and concentrates on the interests of consumers (such as economic cost and health) to make consumers more inclined to consider personal interests (Schuhwerk and Lefkoff-Hagius, 1995). Moreover, scholars have found that it is best to highlight only one kind of interest appeal in the advertising of green products, which means that we should not mix self-interest and altruism in order not to affect the promotion effect (Feiler et al., 2012).

### The Matching Effect of Brand Anthropomorphic Image Type and Advertising Appeals

Existing studies have shown that information processing fluency can, indeed, have a positive impact on the cognition of consumers, including the evaluation of products and brands. The stronger the perceived fluency is, the more likely it is to produce a better evaluation (Lee and Labroo, 2004; Labroo and Rucker, 2010). In a similar vein, proper matching between the various elements (symmetry, color, and background) in the promotion of advertising can enhance consumer information processing fluency, and proper matching can promote the fluency of consumer information processing and enhance consumers’ attitude towards advertising (Reber et al., 2004).

Based on the theory of information processing fluency, consumers will search for relevant clues to prove that brands can meet their interests when they come into contact with self-interest advertising demands. When the brand anthropomorphic image is cool, consumers who perceive cool perception will consider that the product will bring some benefits to themselves, such as hedonic or functional value, and even help them realize themselves (Holbrook and Batra, 1987; Sundar et al., 2014; Im et al., 2015), thus aligning with the content of self-interested advertising. When consumers are exposed to altruistic advertising appeals, they will subconsciously look for clues to prove that brands benefit society and help others. If the brand anthropomorphic image is cute at this time, the initiation of cute perception will induce consumers to infer positively that the brand is kind, warm, and honest (Keating et al., 2003; Gorn et al., 2008; Windhager et al., 2008). These characteristics are often highly correlated with prosocial behavior; thus, the cute brand anthropomorphic image and altruistic advertising appeal are more consistent. If the advertising appeal of the product and the anthropomorphic image match accurately, it will improve the perception information fluency of the consumer and reduce the perceived risk in ways of increasing the trust in the brand and then improve the purchase intention. Taken together, we propose the following hypotheses:

Hypothesis 1. Advertising appeal type and brand anthropomorphic image type interact to affect the willingness of consumers to buy green products.

Hypothesis 1a. For consumers who are exposed to self-interest advertising appeals, a cool brand anthropomorphic image (vs. cute) can stimulate a higher willingness to buy green products.

Hypothesis 1b. For consumers who are exposed to altruistic advertising appeals, a cute brand anthropomorphic image (vs. cool) can stimulate a higher willingness to buy green products.

### The Mediating Role of Brand Trust

Studies have shown that consumers often lack trust in advertising that claims to be green and they tend to doubt the authenticity and motivation of their environmental advocacy, which hinders them from further adopting green consumption behaviors (Tung et al., 2012). Fournier (1996) suggested that brand trust is the degree of confidence and dependence on brands of consumers. Hess (1995) divided brand trust into three dimensions: altruism, sincerity, and reliability. According to the peculiarities of the trusted side, McAllister (1995) and Mayer et al. (1995) put forward three influencing factors of trust, namely capacity, goodwill, and integrity. Capacity is a set of skills, abilities, and characteristics that performs tasks professionally and have a large influence in a certain field. Goodwill is the degree to which the trusted side is likely to do a good deed (the motivation for doing good is not a self-centered interest), while altruism contributes to the degree of goodwill trust. Integrity is the belief that the trusted side will follow a set of established principles. Each dimension of brand trust is independent of each other and can be separate and act as an independent variable (McAllister, 1995), which means that consumers may have different brand trust due to different properties of the brand. According to the traits of cuteness and coolness, this study chooses goodwill trust and capacity trust to explain the matching effect between brand anthropomorphic image type and advertising appeal type. Specifically, brand goodwill trust means that consumers believe that brands are not just profit oriented but altruistic as well. Brand ability trust refers to consumers who believe that the brand has certain competence and technology to produce products with specific functions to meet the interests of individuals.

Corresponding to the combination of different brand anthropomorphic images and advertising appeals, the more specific hypotheses of this study are derived as follows. Self-interested appeals provide consumers with utilitarian motives (Lantos, 2015), which will lead consumers to pay more attention to the efficacy of green products when evaluating them. The exclusive value that the cool anthropomorphic image of brands can provide (such as hedonic benefit or practical benefit) is more consistent with the self-interested advertising appeal, which enables consumers to process information more fluently and have a higher perception of the authenticity of the product information so that consumers are more likely to trust in the ability of a brand and believe that the brand can truly bring them the benefits promised in the advertising appeal. On the other hand, Sherman and Haidt (2011) pointed out that the reaction of people to cuteness is a moral emotion. Compared with the cool brand anthropomorphic image, which makes consumers feel rebellious, highly independent and mature, the cute brand anthropomorphic image has a higher internal consistency with the altruistic advertising appeal. In a similar vein, consumers would be more fluent in information processing and more inclined to believe that the brand of cute anthropomorphism will truly fulfill the altruistic content in advertising to care for the welfare of the whole society, which result in greater brand goodwill trust and higher willingness to adopt the green products. Taken together, the following hypotheses are proposed:

Hypothesis 2. Brand trust mediates the interactive effect of brand anthropomorphic image type and green product advertising appeals on green product purchase intention.

Hypothesis 2a. Brand capacity trust mediates the interaction of cool brand anthropomorphic image and self-interest advertising appeals on the purchase intention of consumers of green products.

Hypothesis 2b. Brand goodwill trust mediates the interaction of cute brand anthropomorphic image and altruistic advertising appeals on the purchase intention of consumers of green products.

## Study 1: The Interaction Effect of Brand Anthropomorphic Image Type and Advertising Appeals on Green Product Purchase Intentions

Study 1 is mainly to test whether the type of brand, anthropomorphic image, and advertising appeals interact to influence the purchase intention of consumers of green products, namely H1.

### Pretest

**FIGURE 2:**
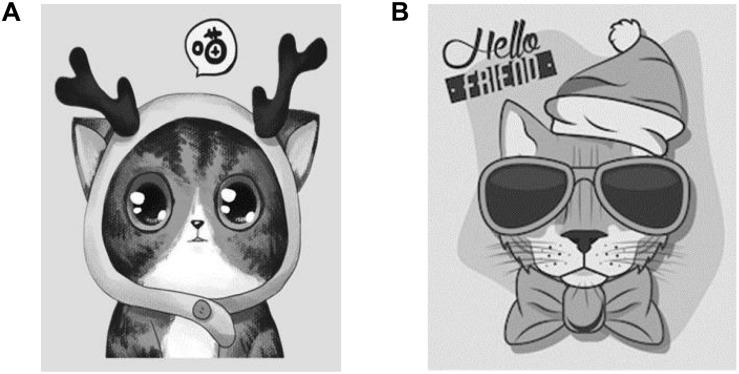
Brand anthropomorphic images of cuteness **(A)** and coolness **(B)** in Study 1.

Before the study, the appropriate brand anthropomorphic images (cool vs. cute) were selected as the experimental materials through the pretest. The cute brand anthropomorphic image selected a cat with a round face and big eyes as shown in Figure 2A (Gorn et al., 2008; Wang et al., 2017), which added tautology and a modal particle into the description of advertising to better initiate cute perception (Argo et al., 2010). The cool anthropomorphic image was a cat with dark glasses and a neutral expression as shown in Figure 2B (Wang et al., 2014; Warren et al., 2018). Autonomous and rebellious descriptions were added in its advertising material to initiate the perception of coolness (AS Belk et al., 2010; Warren and Campbell, 2014).

This pretest recruited participants from Credamo, a Chinese online group similar to Mturk. Thirty-five participants from different backgrounds completed the survey (men, 54.3%; women, 45.7%, M_age_ = 22.74). The measurement of cute perception drew on Nenkov and Scott’s scales (three items: the brand image is cute/the brand image is adorable/the brand image is endearing, α = 0.817) (Nenkov and Scott, 2014). The measure of cool perception referred to the scales proposed by Sundar et al. (2014) and Warren et al. (2019), which mainly measures five dimensions of autonomy, rebelliousness, complexity, originality, and vitality(five items: the brand image is mature and capable/the brand image is dared to break the convention/the brand image will act following their own will/the brand image is full of vitality/the brand image is creative, α = 0.890); the above scales were all seven-point scales. The results show that the cool perception of the cool anthropomorphic image was significantly higher than that of cute anthropomorphic image (M_cool_ = 4.824 vs. M_cute_ = 3.367; *t* = 8.016, *p* < 0.001, *d* = 2.834); the cute perception of the cute anthropomorphic image was significantly higher than that of the cool anthropomorphic image (M_cute_ = 4.907 vs. M_cool_ = 3.353; *t* = –6.634, *p* < 0.001, *d* = 2.345). The above results indicated that the type of brand anthropomorphic image is successfully manipulated, and these materials could be used in further experiments.

### Experimental Design and Subjects

A between-subject design of 2 (brand anthropomorphic images: cute vs. cool) × 2 (advertising appeals: altruistic vs. self-interested) was used in Study 1, and the participants were randomly assigned to one of the four groups. The participants were recruited on the questionnaire platform Credamo by offering cash rewards. One hundred four valid questionnaires were received (men, 47.1%; women, 52.9%; M_age_ = 23.08).

### Experimental Procedures

First, the participants were required to imagine meeting advertising for an environmentally friendly water cup made of wheat straw when shopping online. The anthropomorphic images selected in the pretest were used as brand logos in the advertising, and the specific manipulation was the same as the pretest. The manipulation of advertising appeals referred to the design of Kareklas et al. (2014) and Yang et al. (2015). The altruistic advertising appeals group mainly expressed that the use of environment-friendly water cups was conducive to protecting the nature and ecological environment, specifically the contents of the altruistic group are as follows: “The water cup is made of wheat straw, natural and non-toxic, ecologically degradable, environmentally friendly, and pollution free. Come and protect the lovely nature.” While the self-interested advertising appeals group highlighted the impact of environment-friendly water cups on consumer health, the specific contents are as follow: “The water cup is made of wheat straw, healthy and non-toxic, the wheat fragrance is thick, and the ecology is safe. Let us drink to the health.” The length of all advertising words in Study 1 was balanced to avoid unnecessary interference.

Second, the participants were required to rate brand anthropomorphic images on cute and cool scales as a manipulation test. Then, the participants needed to rate perceived self-interest (three items: advertising content based on environmental protection considerations/resource conservation considerations/social overall interest considerations, α = 0.833) and perceived altruism (three items: advertising content based on personal health considerations/personal use considerations/personal interest considerations, α = 0.868) of advertising appeals (Kareklas et al., 2014). The participants reported their willingness to purchase the green product (1 = not at all probable, 7 = very probable; α = 0.846) (Tezer and Bodur, 2019). The above-mentioned measurement scales are all seven-point scales and have been adjusted and adapted to the situation of this experiment.

Finally, the participants also completed the relevant test items in consideration of the influence of brand familiarity on purchase intention and demographic messages (Campbell and Keller, 2003).

## Results

### Manipulation Check

The first is to test the manipulation effect of brand anthropomorphic type. *T*-test results showed that the cute perception of the cute anthropomorphic image was significantly higher than that of the cool anthropomorphic image (M_cool_ = 2.969 vs. M_cute_ = 5.405; *t* = –21.269, *p* < 0.001, *d* = 4.211); the cool perception of the cool anthropomorphic image was significantly higher than that of the cute anthropomorphic image (M_cool_ = 5.438 vs. M_cute_ = 3.055; *t* = 18.839, *p* < 0.001, *d* = 3.731), indicating that the manipulation of the anthropomorphic image is successful and then conducted a *t*-test on the manipulation of advertising appeals; the results showed that the perceived altruism of the altruistic advertising appeal group was significantly higher than that of the self-interested advertising appeal group (M_altruistic_ = 4.660 vs. M_self__–interested_ = 3.327; *t* = –12.092, *p* < 0.001, *d* = 2.395), and the perceived self-interested of self-interested advertising appeal group was significantly higher than that of the altruistic advertising appeal group (M_self__–interested_ = 4.566 vs. M_altruistic_ = 3.379; *t* = 11.955, *p* < 0.001, *d* = 2.367), indicating that the manipulation of advertising appeals is successful.

### Hypothesis Test

**FIGURE 3:**
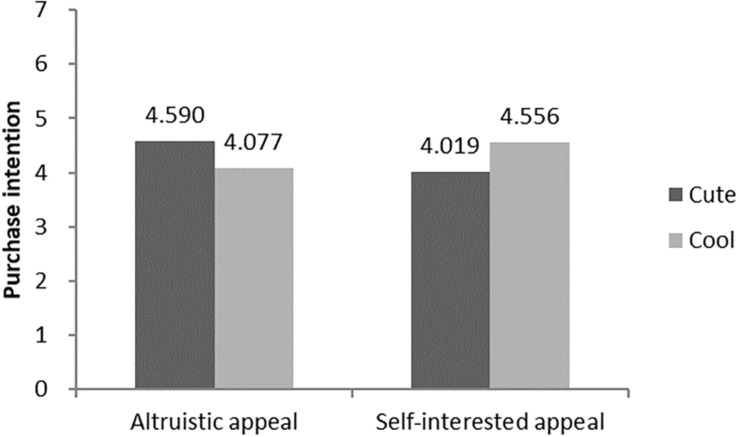
The interaction of brand anthropomorphic images and advertising appeals.

Firstly, an analysis of variance (ANOVA) with the brand anthropomorphic image type and advertising appeals as independent variables and purchase intention of green products as the dependent variable revealed a significant interaction effect (F = 40.610, *p* < 0.05, η^2^ = 0.289, see Figure 3). Further analysis showed that, in the altruistic appeal condition, the participants were more likely to buy green products with cute anthropomorphic images (M_cool_ = 4.077 vs. M_cute_ = 4.590; F = 19.049, *p* < 0.001). In contrast, in the self-interested appeal condition, the participants were more willing to buy green products with cool anthropomorphic images (M_cool_ = 4.556 vs. M_cute_ = 4.019; F = 21.631, *p* < 0.001).

Next, we added brand familiarity for covariance analysis. It revealed the interaction between the advertising appeals and brand anthropomorphic image type on the purchase intention of green products was still significant (F = 40.081, *p* < 0.001, η^2^ = 0.288), indicating that the influence of brand familiarity on the matching effect can be excluded. Therefore, H1, H1a, and H1b were supported in Study 1.

### Discussion

Study 1 investigated the interaction effect of advertising appeals and brand anthropomorphic images on purchase intention of green products, and successfully verified H1a and H1b. However, Study 1 still has some deficiencies, such as the relationship between the brand anthropomorphic image and the product is not strong enough, and the gender perception of different brand anthropomorphic images is not taken into account. Actually, some scholars suggested that cuteness is often associated with feminine designs that may affect the robustness of experimental results (Locher, 2007; Moeran and Skov, 2013), for instance, using the favorite colors or styles of females.

In view of this, different product categories (environmentally friendly juice) were used to design brand anthropomorphic images to further strengthen the connection between brand anthropomorphic images and products, and the interference of perceived gender was excluded in Study 2. In Study 2, the robustness of the experimental results was also enhanced by adding a choice situation, that is, asking consumers to choose the products represented by different brand anthropomorphic images in different groups of advertising appeal types, and the mediating role of two kinds of perceived brand trust (goodwill trust and capacity trust) was verified in Study 2.

## Study 2a. The Interaction Effect of Brand Anthropomorphic Image Type and Advertising Appeals on Green Product Purchase Intentions

Study 2 adopted a new green product category (juice) and redesigned the brand anthropomorphic images (orange) that were different from Study 1 to strengthen the linkages between images and products. Meanwhile, Study 2 also excluded the interference of gender perception to enhance the robustness of the experimental results. In addition, Study 2a did not adopt the measurement method of purchase intention used in Study 1. Instead, the consumers were asked to make a choice between two green products with anthropogenic images to measure their purchase intention.

### Pre-test

**FIGURE 4:**
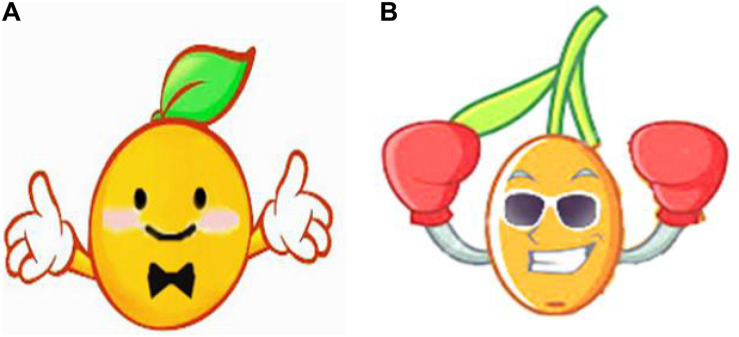
Brand anthropomorphic images of cuteness **(A)** and coolness **(B)** in Study 2.

The anthropomorphic image selected a sort of fruit (orange) as the object to ensure it could be well connected with the green products (juice). As for image design, the colors of both anthropomorphic images are orange, and the character posture, accessories and painting styles are similar as well (see Figure 4). The design of advertising text materials still followed the information framework that has been adopted in Study 1, except that it was conveyed in the first person to enhance the anthropomorphic effect.

The pretest obtained 34 valid questionnaires from Credamo (men, 52.9%; women, 47.1%, M_age_ = 22.5). The results showed that the cool perception of the cool anthropomorphic image was significantly higher than that of the cute anthropomorphic image (M_cool_ = 4.459 vs. M_cute_ = 3.424; *t* = –6.604, *p* < 0.001, *d* = 2.335). Likewise, the cute perception of the cute anthropomorphic image was significantly higher than that of the cool anthropomorphic image (M_cute_ = 4.824 vs. M_cool_ = 3.412; *t* = –7.155, *p* < 0.001, *d* = 2.53). Since cute anthropomorphic images may be intuitively considered more feminine, and cool anthropomorphic images may be considered more masculine, this may also affect the results of subsequent experiments. However, the results revealed that there was no significant difference in male perception between the two anthropomorphic images (M_cool_ = 4.444 vs. M_cute_ = 3.875; *t* = –1.656, *p* = 0.111 > 0.05), and there was no significant difference in female perception between the two anthropomorphic images as well (M_cool_ = 3.667 vs. M_cute_ = 4.063; *t* = 1.868, *p* = 0.072 > 0.05). Therefore, these materials could be used in Study 2.

### Experimental Design and Subjects

Study 2a recruited participants through the online questionnaire platform Credamo by offering cash rewards. Product selection scenarios were used to measure the willingness of the participants to buy green products. In study 2a, 69 valid questionnaires were received (men, 47.8%; women, 52.2%, M_age_ = 22.75).

### Experimental Procedures

In study 2a, the participants were randomly assigned to one of the two groups: the altruistic advertising appeal group or the self-interested advertising appeal group. For the manipulation of brand anthropomorphic images, the participants were asked to imagine buying juice online and then encountered two advertisements about ecological juice from the northwest ecological orchard. The advertising contained the brand logos, which were the two anthropomorphic images tested in the pretest. More importantly, the product introduction in the advertisement was narrated in the first person to initiate perceptions of cute and cool. Besides, different advertising slogans were used to manipulate the type of advertising appeal (Kareklas et al., 2014; Yang et al., 2015). In the altruistic advertising appeal group, the content of advertising emphasized the contribution of the ecological orchards to control the harsh ecological environment, such as desert, while the self-interested advertising appeal group highlighted that the juice was organic, which is beneficial to the health of individuals. After viewing ads, the participants made a choice about which product they were more willing to buy. Then, the participants answered the manipulation test of the brand anthropomorphic image and the advertising appeal (the scales were the same as Study 1, only a little adjustment according to the context). Finally, the questionnaire measured the demographic variables of the participants, and the scales above were all seven points.

### Results

#### Manipulation Check

In study 2a, the results of the *t*-test revealed that, in the self-interested advertising appeal group, the cool perception of the cool anthropomorphic image was significantly higher (M_cool_ = 5.159 vs. M_cute_ = 3.047; *t* = 14.835, *p* < 0.001, *d* = 5.165). Likewise, the cute perception of the cute anthropomorphic image was significantly higher (M_cute_ = 5.226 vs. M_cool_ = 2.971; *t* = –12.548, *p* < 0.001, *d* = 4.369). In the altruistic advertising appeal group, the results showed that the cool perception of the cool anthropomorphic image was higher (M_cool_ = 5.286 vs. M_cute_ = 3.310; *t* = 21.333, *p* < 0.001, *d* = 7.32), and the cute perception of the cute anthropomorphic image was higher (M_cute_ = 5.324 vs. M_cool_ = 3.267; *t* = –18.602, *p* < 0.001, *d* = 6.38), which indicated that the manipulation of the anthropomorphic image was successful. Next, the manipulation of the advertising appeal was tested. The results indicate that the perceived altruism of the altruistic advertising appeal group was significantly higher than that of the self-interested advertising appeal group (M_altruistic_ = 4.648 vs. M_self__–interested_ = 3.324; *t* = –10.391, *p* < 0.001, *d* = 2.539), and the perceived self-interested of self-interested advertising appeal group was significantly higher than that of the altruistic advertising appeal group (M_self__–interested_ = 4.647 vs. M_altruistic_ = 3.420; *t* = 10.206, *p* < 0.001, *d* = 2.494). The advertisement appeal in Study 2a was successfully manipulated.

#### Hypothesis Test of Interaction Effect

For Study 2a, 20 of the 34 participants in the self-interest advertising appeal group chose the product of the cool brand anthropomorphic image, accounting for 58.8%, and 14 participants chose the product of the cute brand anthropomorphic image, accounting for 41.2%. In the altruistic advertising appeal group, 12 (34.3%) of the 35 participants were willing to choose products with the cool brand anthropomorphic image; the rest of the participants chose the product with the cute brand anthropomorphic image. Chi-square test results showed that the preference for the product with cute or cool anthropomorphic images between the two groups was significant (χ^2^ = 4.176, *p* = 0.041 < 0.05). Specifically, the altruistic advertising appeals group was more likely to choose products with a cute brand anthropomorphic image, while the selfish advertising appeals group preferred to choose products with a cool brand anthropomorphic image. Study 2a proved H1a and H1b again.

## Study 2b. The Mediating Role of Brand Trust

The main purpose of Study 2b was to test the mediating role of perceived brand trust (goodwill trust and capacity trust) providing ways of explaining the results of Study 1.

### Experimental Design and Subjects

Study 2b recruited participants through the online questionnaire platform Credamo by offering cash rewards and 107 valid questionnaires were received in Study 2b (48.6% men, 51.40% women, M_age_ = 23.15).

### Experimental Procedures

A between-subject design of 2 (brand anthropomorphic images: cute vs. cool) × 2 (advertising appeals: altruistic vs. self-interested) was used in Study 2b, and the participants were randomly assigned to one of the four groups. The experimental conditions and variable manipulation methods were the same as those used in Study 2a on the whole. The difference was that, after watching the advertisement, the participants would complete the following five parts rather than making a choice about the products: the purchase intention of green products, manipulation test of brand anthropomorphic image and advertising appeal, brand familiarity, and perceived brand trust. The demographic variables were measured at the end as before. All variables except perceived brand trust were measured by the scales used in Study 1 (adjusted only slightly according to the context). This study drew on the trust scale proposed by Mayer and Davis (1999) to measure brand capacity trust (five items: the brand is capable of producing the product very much/is aware of its product very much/can always achieve its goals/has considerable professional competence/is particularly good at improving consumer health, α = 0.926) and brand goodwill trust (five items: the brand is attentive to others/thinks the needs and expectations of others are important/never intentionally damages the interests of others/thinks it is important to really care for others/will make every effort to help others, α = 0.916).

### Results

#### Manipulation Check

In Study 2b, the cool perception of the cool anthropomorphic image was higher (M_cool_ = 4.513 vs. M_cute_ = 3.496; *t* = 10.314, *p* < 0.001, *d* = 2.013). Also, the cute perception of the cute anthropomorphic image was higher (M_cute_ = 4.636 vs. M_cool_ = 3.264; *t* = –12.092, *p* < 0.001, *d* = 2.36). The anthropomorphic image in Study 2b was successfully manipulated. As for advertising appeals, the perceived altruism of the altruistic advertising appeal group was significantly higher (M_altruistic_ = 4.482 vs. M_self__–interested_ = 3.447; *t* = –9.418, *p* < 0.001, *d* = 1.838), and the perceived self-interest of self-interested advertising appeal group was significantly higher (M_self__–interested_ = 4.465 vs. M_altruistic_ = 3.407; *t* = 11.091, *p* < 0.001, *d* = 2.165). This suggested that the manipulation of advertising appeals in Study 2b was successful.

#### Hypothesis Test of Interaction Effect

**FIGURE 5:**
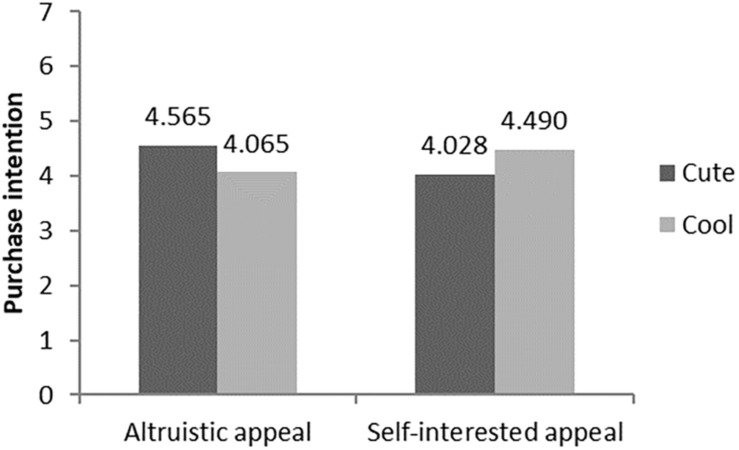
The interaction of brand anthropomorphic images and advertising appeals.

For Study 2b, two-factor ANOVA found that advertising appeals and brand anthropomorphic images had a significant interaction effect on the purchasing intention of green products (F = 54.306, *p* < 0.001, η^2^ = 0.345, see Figure 5). Further analysis indicated that, in the altruistic advertising condition, the participants were more willing to buy green products with the cute brand anthropomorphic image (M_cute_ = 4.565 vs. M_cool_ = 4.065; F = 24.848, *p* < 0.001); when it came to self-interested advertising appeals, the participants had higher willingness to buy green products with the cool anthropomorphic brand image (M_cool_ = 4.490 vs. M_cute_ = 4.028; F = 29.585, *p* < 0.001). Then, we added brand familiarity for covariance analysis. It revealed that the interaction between the advertising appeals and the brand anthropomorphic image on the purchase intention of green products is significant as before (F = 53.629, *p* < 0.001, η^2^ = 0.345). Therefore, H1, H1a, and H1b were supported again in Study 2b.

### The Mediation Analysis of Brand Capacity Trust and Brand Goodwill Trust

Next, we examined the role of brand capacity trust and brand goodwill trust in explaining the observed interaction. First, a mediation analysis with 5,000 bootstrap samples (model 8) with the brand anthropomorphic image as the independent variable, advertising appeal as the moderator, brand capacity trust as the mediator, and purchase intention of green products as the dependent variable was conducted to test H2a (Preacher et al., 2007). The result revealed a significant mediating effect of brand capacity trust when the advertising appeal is self-interested [β = –0.309.95% CI (–0.499, –0.148)]. However, the mediating effect of brand capacity trust was no longer significant once the advertising appeal was altruistic [β = –0.009, 95% CI (–0.100,0.092)]. Therefore, H2a was supported. Similarly, we used the same approach to examine the mediating role of brand goodwill trust; conditional indirect effects supported our predictions: When the advertising appeal was altruistic, the mediating effect of brand goodwill trust was significant [β = –0.112, 95% CI (0.005,0.250)]. However, when the advertising appeal was self-interested, the indirect effect was not significant anymore [β = –0.013, 95% CI (–0.013,0.118)], indicating that H2b was supported.

### Discussion

Study 2 modified the stimulus material to increase the validity and applicability of experimental findings. Under different product types (ecological juice) purchase scenarios, the matching effect of brand anthropomorphic images and advertising appeals was demonstrated again through two sub-experiments in Study 2. Moreover, Study 2 further investigated the underlying mechanism of the matching effect. Study 2b particularly confirmed the mediating role of brand trust in the matching effect. Specifically, brand capacity trust mediated the interaction of the cool brand anthropomorphic image and self-interest advertising appeals on the purchase intention of green products of consumers, whereas brand goodwill trust mediated the interaction of cute brand anthropomorphic image and altruistic advertising appeals on the purchase intention of green products of consumers.

## Conclusion and Discussion

### Conclusions

The current research advances our understanding of how brand anthropomorphic images (cute/cool) and advertising appeals (self-interested/altruistic) affect the purchase intention of green products of consumers. Across two experiments, we found that there was a matching effect between brand anthropomorphic images and advertising appeals. To be specific, when using self-interested advertising appeals, consumers prefer green products with a cool anthropomorphic image. When it comes to altruistic advertising appeals, consumers are more inclined to purchase green products with a cute anthropomorphic image. Furthermore, we repeated the effect found in Study 1 under different experimental contexts and explored the potential mechanism of the matching effect by examining the mediating roles of brand capacity trust and brand goodwill trust as well. For self-interested advertising appeals, the cool anthropomorphic image of the brand (vs. cute) can lead to higher brand capacity trust, thus stimulating higher purchase intention of green products. As for altruistic advertising appeals, the cool anthropomorphic image of the brand (vs. cute) can result in higher brand goodwill and trust in ways of improving the willingness of consumers to buy green products.

### Theoretical Implication

The current research provides several theoretical implications. First, this research enriches the literature on anthropomorphism in the field of green consumption. At present, studies have investigated the impact of anthropomorphism on the beneficiaries of green consumption behavior (such as earth, nature, forest, animals, etc.) on environmental protection behavior, including green consumption behavior. Few studies have focused on how anthropomorphic products or brands affect the willingness to buy green products. Recent research on green products has hinted at the possibility that different anthropomorphic images may play different roles in product purchase and other consumption behaviors (Puzakova et al., 2013; Puzakova and Kwak, 2017; Reavey et al., 2018). In the field of environmental protection and green consumer behavior, more studies examined the impact of the existence or absence of anthropomorphic on green consumer behavior (Tam et al., 2013; Ahn et al., 2014; Williams et al., 2015; Han et al., 2019; Zhu et al., 2019). However, the literature about the effect that different types of anthropomorphic images have different effects on green consumption behavior is still limited. This study mainly discusses the impact of two different brand anthropomorphic images on green consumption, focusing on the anthropomorphism of the green product brand itself rather than the beneficiaries of green consumption (nature, earth, etc.). Specifically, the current research presents an effect of the cute and cool brand anthropomorphic images on the purchase intention of green products under different types of advertising appeal.

Secondly, this research contributes to the literature of purchasing decisions of green consumption. Same as the general consumption behavior, the green consumption behavior includes the following stages as well: generating needs, collecting information, evaluating plans, making decisions on purchase, and post-purchase behavior. Previous studies mostly concentrated on post-purchase behaviors of green consumption, such as disposal and recycling (Tam et al., 2013; Ahn et al., 2014; Han et al., 2019; Ketron and Naletelich, 2019), which are different from the purchasing behaviors of green products concerned in this paper. The current study extends research on green consumption by moving the focus to the stage of making a purchase decision. Purchasing green products is a kind of prosocial behavior in terms of the self-interest attribute and altruistic attribute simultaneously existing in the green products (Mazar and Zhong, 2010). In view of this, consumers tend to trade-off between self-interest and altruistic attributes of green products, which are clearly influenced by marketing context factors, such as anthropomorphism. Therefore, it is worth discussing what effect anthropomorphic factors will have on the specific behaviors of green product purchases. Thus, this research particularly explored how the anthropomorphic image of a brand and advertising appeals promote consumers to make purchasing decisions of green products.

Thirdly, this paper extends the research of different types of anthropomorphism for the field of marketing. A study about anthropomorphism has divided brand anthropomorphism into two types: warmth and competence according to the stereotype model (Aaker et al., 2010). If consumers consider the efficacy of products, the competent brand anthropomorphic image would make consumers have a better evaluation and purchase intention, while the warm brand anthropomorphic image was preferred when consumers had strong attribution needs (Aaker et al., 2010). The research that divided brand anthropomorphic images into partner and servant found that consumers have different image preferences and interaction rules in different situations (P and L, 2012; Alvarez and Fournier, 2016). The current research divided the brand anthropomorphic image into cool and cute and further found that these two types of the anthropomorphic image can play a better role in the marketing and promotion of green products only when they match appropriate advertising appeals.

Finally, this research also enriches the literature on the influencing factors of green consumption behavior. Previous pieces of research on green consumption focused on how to reduce or even eliminate the attitudes and behavior inconsistency of consumers in green consumption behavior through their internal and external factors. Internal factors include positive or negative emotions (Young et al., 2009; Kanchanapibul et al., 2014), degree of environmental concern of consumers (Wang et al., 2014; Zhao et al., 2014), consumer values (Young et al., 2009; Chen and Lobo, 2012; Eze and Ndubisi, 2013; Wang et al., 2014), external factors, such as advertising messages (Sheehan and Atkinson, 2012; Kareklas et al., 2014), advertising appeals (Green and Peloza, 2014; Yang et al., 2015), and product type (Luchs et al., 2010). Recent research on green consumption has hinted at the possibility that brand-attributes products, such as brand-perceived value, brand trust, and brand awareness, may have a positive influence on the purchase intention of consumers (Rahbar and Abdul Wahid, 2011; Zabadi et al., 2016; Ranjan and Kushwaha, 2017). Different from these studies, we mainly investigated the influence of brand anthropomorphism as a marketing communication element on green consumption. Specifically, this research examines the impact of matching different types of brand anthropomorphic images with other approaches of communication (advertising appeal) on green consumption. Therefore, in terms of exploring the relationship between brand attributes and purchasing intention of green products, the content of this research can provide some new inspirations.

### Practical Implication

This study also provides some enlightenment for the practice of green marketing. First of all, the current research provides more choices for the brand image design of enterprises producing green products. In addition to the common competence (vs. warmth) and servant (vs. partner) anthropomorphic image categories, enterprises can also choose to adopt the cool brand anthropomorphic image or the cute brand anthropomorphic image according to their own needs to improve the purchase intention of consumers.

Secondly, in the green marketing promotion, when enterprises use the cool or cute brand anthropomorphic image to attract the attention of consumers, they also need to pay attention to the correct combination of brand images and advertising appeals to achieve a better marketing communication effect. If an enterprise adopts a cute brand anthropomorphic image, it can highlight more altruistic aspects of green products in the design of advertising appeals, improve the goodwill trust of consumers in the brand to achieve a better promotion effect. And if the enterprise chooses to show the cool brand anthropomorphic image, then, in the design of advertisement appeal, it can highlight more self-interested aspects of green products and improve the capacity trust of consumers in the brand, which can also make the promotion effect better.

Finally, this research is of great significance for those green product enterprises whose environmental protection propositions are questioned by consumers to enhance brand trust. Studies have found that even consumers with a high degree of environmental concern are hesitant to buy green products because they tend to doubt the authenticity of green claims of enterprises (Nittala, 2014). According to the influence of information fluency on perceived authenticity, the better the information fluency, the more likely consumers are to believe the authenticity of the information, the lower the perceived risk, and the greater the brand trust. On the contrary, consumers will doubt the authenticity of information and have a low level of brand trust, which will hinder the occurrence of purchasing behaviors (Reber et al., 1998; Lau and Lee, 1999). Therefore, enterprises whose environmental protection claims are questioned can consider improving the matching degree between advertising claims and the brand image in the marketing communication to enhance brand trust. For example, combining with altruistic advertising appeals when choosing a cute brand anthropomorphic image can effectively improve brand goodwill trust of consumers to reduce doubts of consumers about the environmental protection propositions of enterprises.

### Limitation and Future Research Direction

There are still some limitations in this research. First of all, the context factors of green consumption are complex; some of them may influence the results but are not taken into account. For example, from the perspective of an individual, environmental protection concepts, values, self-construction, and other personal factors of consumers may be added to this study as moderator variables. Some external factors, such as communication, advertising framework, the level of specificity of advertising (vs. abstraction), the type of product (self-interest vs. altruism, pleasure vs. utility, durable vs. expendable), and even the price level of the product may also play a moderating role, which could be explored in future research.

Second, the mediation mechanism can be further demonstrated. Due to the space limitation of this paper, only brand trust is selected as the mediating variable in the research process, but there may be other intermediaries, such as the concern of consumers for the environment. Thus, we can test whether there are other mediation mechanisms in research and then provide corresponding insights for the further development of green consumption.

Lastly, whether there are different influences on green consumption between other anthropomorphic types and the anthropomorphic images of current research remains to be investigated. This research did not compare the two brand anthropomorphic images of competence and warmth with the two types of brand anthropomorphic images proposed, but it is noteworthy that they are similar to a certain extent. It is expected that warmth (the degree of cuteness is low, but consumers can still perceive warmth) may not have such a strong effect. Even matching altruistic advertising appeals may not easily lead to a stronger perception fluency of consumers, thus leading to higher brand trust; whereas the effect of the competent brand anthropomorphic image may be similar to the cool brand anthropomorphic image. These all need to be verified by future research.

## Data Availability Statement

The original contributions presented in the study are included in the article/Supplementary Material, further inquiries can be directed to the corresponding author/s.

## Ethics Statement

The studies involving human participants were reviewed and approved by the Ethics Committee of the School of Management, Jinan University, China. The patients/participants provided their written informed consent to participate in this study. Written informed consent was obtained from the individual(s) for the publication of any potentially identifiable images or data included in this article.

## Author Contributions

SY and YLu conceived and designed the experiments. SY and YLi carried out the experiments, analyzed the experimental results, and wrote the manuscript. LT edited the manuscript. All authors contributed to the article and approved the submitted version.

## Conflict of Interest

The authors declare that the research was conducted in the absence of any commercial or financial relationships that could be construed as a potential conflict of interest.

## Publisher’s Note

All claims expressed in this article are solely those of the authors and do not necessarily represent those of their affiliated organizations, or those of the publisher, the editors and the reviewers. Any product that may be evaluated in this article, or claim that may be made by its manufacturer, is not guaranteed or endorsed by the publisher.
